# Self-report of domestic violence and forced sex are related to sexual risk behaviors in a sample of juvenile detainees

**DOI:** 10.1186/s40352-020-00116-4

**Published:** 2020-06-23

**Authors:** Lea Selitsky, Norman Markowitz, Dwayne M. Baxa, Linda Kaljee, Cheryl A. Miree, Nishat Islam, Chez Burse, Rehnuma Newaz, Doreen Dankerlui, Gordon Jacobsen, Christine Joseph

**Affiliations:** 1 Internal Medicine, Johns Hopkins Hospital, Baltimore, USA; 2 Division of Infectious Diseases, Henry Ford Health System and School of Medicine, Wayne State University, Detroit, USA; 3 William Beaumont School of Medicine, Oakland University, Rochester, USA; 4 Division of Infectious Diseases, Henry Ford Health System, Detroit, USA; 5 Global Health Initiative, Henry Ford Health System, Detroit, USA; 6 Department of Public Health Sciences, Henry Ford Health System, 1 Ford Place, Detroit, Michigan USA

**Keywords:** Justice-involved youth, Sexual risk behaviors, Trauma, Substance use, Juvenile, Domestic violence

## Abstract

**Background:**

Justice-involved youth have higher rates of sexually transmitted infections (STIs), and a higher prevalence of the associated sexual risk behaviors. Sexual risk behaviors are also associated with alcohol and drug use. Research suggests that a history of trauma is an important predictor of alcohol and drug use in youth offenders, and therefore is a likely contributor to sexual risk behavior in this population. The objective of this analysis is to determine the association of trauma, specifically, domestic violence and forced sex, to six sexual risk behaviors and a history of chlamydia among detained youth.

**Methods:**

The analysis uses data from a convenience sample of detainees assenting to HIV testing conducted December 2016 – August 2017 using the state-certified Voluntary Counseling Testing and Referral (VCTR) process.

**Results:**

Of the 379 youth that received VCTR at the facility, 308 (81.3%) were used in this analysis. Report of domestic violence was significantly associated with sex under the influence of alcohol and was also significantly associated with sex under the influence of marijuana. Forced sex was associated with a sexual partner of unknown HIV status.

**Conclusions:**

Traumatic experiences were related to sexual risk behaviors in this analysis, and substance use was strongly implicated in the association. Trauma is known to be a catalyst to sexual risk behaviors, substance use, and delinquency in adolescence. Results support the findings of other investigators and re-iterate the need for trauma-informed interventions that can improve the life trajectories of detained youth.

## Background

### Sexually transmitted infections and justice-involved youth

Every day, approximately 48,000 youth in the US are held in facilities away from home as a result of justice involvement (Sawyer & Wagner, 2019). African American and Latinx youth from low-income, low resource, and segregated communities are disproportionately represented among justice-involved youth (Sawyer & Wagner, 2019). The lack of resources in the communities in which many justice-involved youth live can result in a lack of exposure to health services and health education, likely contributing to the disparities observed in reproductive health outcomes for this group (Aalsma et al., 2011; Barnert, Perry, & Morris, 2016; Pyle, Flower, Fall, & Williams, 2016). Higher than average rates of sexually transmitted infections (STIs) among justice-involved youth, and the behaviors associated with them, are well-documented (Aalsma et al., 2011; Leve, Van Ryzin, & Chamberlain, 2015; Morris et al., 1995; Spaulding et al., 2013). According to CDC, rates for chlamydia positivity among youth offenders is 14.8% and 6.6% respectively for males and females (Spaulding et al., 2013), and gonorrhea positivity among female offenders is estimated at almost 4% (Barnert et al., 2016; Leve et al., 2015; Spaulding et al., 2013). This is in sharp contrast to the chlamydia and gonorrhea rates of 2.1% and less than 1%, respectively, in the general population of youth aged 15–24 years (Centers for Disease Control and Prevention, 2019). Based on the literature, this trend has continued for over 25 years (Barnert et al., 2016; Dembo et al., 2017; Gillman, Yeater, Feldstein Ewing, Kong, & Bryan, 2018; Hatcher, King, Evans, & Summers, 2017).

Compared to non-offenders, youth offenders are more likely to report behaviors that heighten the risk of STI, including unprotected sex and sex with partners of unknown HIV status (Morris et al., 1995). Behaviors that put youth at risk of acquiring STIs are also associated with alcohol and drug use (Schmiege & Bryan, 2016) and chronic drug users are especially susceptible (Dembo et al., 2019). Gillman et al. (2018) found that use of both marijuana and alcohol or alcohol alone had a stronger association with sexual risk behaviors than using marijuana alone (Gillman et al., 2018), but reports also suggest that alcohol and marijuana are frequently used in combination (Banks et al., 2019; Gillman et al., 2018; Tolou-Shams, Harrison, Hirschtritt, Dauria, & Barr-Walker, 2019). Chronic use of alcohol and binge drinking also have an elevated risk of acquiring HIV (Dembo, DiClemente, Brown, & Faber, 2016; Scott-Sheldon, Carey, Cunningham, Johnson, & Carey, 2016).

The literature on the effect of marijuana use on sexual risk behavior is substantial (Dembo, Faber, et al., 2017; Schumacher, Marzell, Toepp, & Schweizer, 2018; Stephens & Allen, 2018), although not all results show a significant association (Hensel, Stupiansky, Orr, & Fortenberry, 2011). In one meta-analysis, an association between sex under the influence of marijuana was significantly associated with not using a condom, but only for adolescents (Schumacher et al., 2018). Tolou-Shams et al. (2019) reviewed 46 studies on substance use and HIV among justice-involved youth, both cross-sectional and longitudinal (Tolou-Shams et al., 2019). Results of the cross-sectional studies confirmed an association between substance use and sexual risk behaviors but results from longitudinal studies seemed to emphasize the need to consider contextual factors, such as trauma and violence, as drivers of the association (Tolou-Shams et al., 2019).

Recent studies suggest that depression, violence, and trauma contribute to the interrelationship of drug use and sexual risk behavior among justice-involved youth (Clements-Nolle, Larson, Buttar, & Dermid-Gray, 2017; Dembo et al., 2018; Dembo, Faber, et al., 2017). Many justice-involved youths are exposed to painful childhoods and face multiple challenges including family dysfunction, victimization, and community violence (Dembo et al., 2018). According to the National Child Traumatic Stress Network, up to 90% of justice-involved youth have been exposed to trauma or have experienced a traumatic event, with 62% having experienced trauma within the first 5 years of life (Dembo et al., 2018; Dierkhising et al., 2013). Conceptually, a link between trauma exposure and behaviors that increase the risk of STI is well-supported for justice-involved youth (Scott, Duell, & Steinberg, 2018). Research has found that the social and environmental context of the youth’s lived experience (e.g., poverty, community violence, antisocial peers) is more predictive of behavior than calculated propensity scores designed to predict an individual’s inherent inclination to take risks (Scott et al., 2018). Experiencing trauma in early life creates a heightened vulnerability to repeated trauma and victimization (McLaughlin et al., 2013). This is supported by results of a study in which the adverse childhood experiences (ACEs) were assessed in over 64,000 justice-involved youth. ACEs included childhood abuse (physical, emotional, and sexual), neglect (physical and emotional), and household dysfunction (family violence, family substance use, family mental illness, separation/divorce, and family incarceration) (Baglivio, 2016; Baglivio et al., 2014). In that study, 82% of youth experienced at least one ACE, and 67% of these were exposed to an additional 4–6 ACES (Baglivio, 2016). Investigators reported that very rarely do justice-involved youth experience ACEs as isolated events, and that these exposures have cumulative effects (Baglivio, 2016; Baglivio et al., 2014).

The literature supports exposure to adversity in justice-involved youth as an important factor along the causal pathway to sexual risk behaviors shown to be related to acquiring STIs ((K. Clements-Nolle et al., 2017; Conrad, Queenan, Brown, & Tolou-Shams, 2017; Seth, Jackson, DiClemente, & Fasula, 2017). To our knowledge, there are five studies in the last 5 years that specifically focus on the association between various forms of trauma and sexual risk behavior among juvenile detainees (K. Clements-Nolle et al., 2017; Fasula et al., 2018; Leve et al., 2015; Naramore, Bright, Epps, & Hardt, 2017; Seth et al., 2017) (although Fasula and Seth use the same study population). Four of the five studies bring issues of female youth incarceration to the forefront by focusing only on female detainees (K. Clements-Nolle et al., 2017; Fasula et al., 2018; Leve et al., 2015; Seth et al., 2017). Not included in this count are Moreland et al. (2018) who used a nationally representative sample of females (not detainees Moreland et al. (2018), as well as Negriff (2018), and Thompson et al. (2017) who used populations from Child Family Services and Child Protective Services, respectively (Negriff, 2018; Thompson et al., 2017). Seth showed that community trauma in Atlanta, Georgia, was associated with several sexual practices (e.g., unprotected sex, justice-involved sex partner, and marijuana use) in a sample of 188 female detainees aged 13–17 years (Seth et al., 2017). Later, using the same population, Fasula reported that psychological abuse (emotional abuse, pregnancy coercion) and violence (physical and sexual abuse) were associated with sexual risk behaviors and STIs (Fasula et al., 2018). Two of these four studies examined potential pathways. Leve et al., 2015, in a study including 166 female juvenile offenders in Oregon, reported that level of comfort in discussing safe sex practices with partners during adolescence modified the relationship between sexual abuse (physical and sexual abuse) during childhood and unsafe sexual practice in young adulthood (Leve et al., 2015). Clement found that alcohol/drug use, psychological distress and dating violence were mediators of the relationship between childhood maltreatment (physical abuse, sexual abuse, supervision neglect) and unprotected sex(Clements-Nolle et al., 2017). While sample sizes for these studies were relatively small and restricted to females, and although findings were comparatively consistent, we believe the literature is not yet saturated with research on trauma and its association to sexual risk behavior among juvenile detainees, both males and females.

A critical part of prison reform is the call for evidence-based, trauma-informed strategies aimed at providing more support to detained youth, the staff that care for them, their families, and the community (Branson, Baetz, Horwitz, & Hoagwood, 2017; Scott et al., 2018; Skinner-Osei, Mangan, Liggett, Kerrigan, & Levenson, 2019). A greater understanding of how selected traumatic life events reported by justice-involved youth impact sexual risk behavior can inform the development of interventions targeting this population. The objective of this analysis is to add to the current literature by assessing the relationship between report of previous forced sex or domestic violence and sexual risk behaviors or a history of chlamydia in a convenience sample of male and female juvenile detainees who assented to HIV testing.

## Methods

### Study setting

This study was conducted in the Wayne County Juvenile Detention Facility (JDF) located in the metropolitan area of Detroit, MI. The JDF has the capacity to house up to 194 youth aged 9–20 years awaiting criminal adjudication, sentencing, or placement. In 2017, the JDF admitted 1294 youth. All youth receive a medical evaluation as part of facility intake, including a screen for sexually transmitted infections. HIV Voluntary Counseling Testing and Referral (VCTR) is performed at the facility by local organizations. Henry Ford Health System (HFHS) investigators expanded the JDF’s VCTR program in the context of a larger randomized controlled trial aimed at the development and evaluation of a web-based strategy to increase community-based access to HIV/STD testing, treatment, and risk reduction education.

### Voluntary counseling testing and referral and data collection

In Michigan persons 13 years of age or older may be HIV tested without the requirement of parental consent (Centers for Disease Control and Prevention, 2018). For detained minors the JDF Medical Director holds medical power of attorney. Research staff approached JDF residents for VCTR during the period of December 2016 through October 2017. For those assenting to VCTR, point of care testing was performed using the Alere Determine™ HIV-1/2 Ag/Ab Combo rapid test (Orgenics, Ltd., Yavne, Israel). State-certified HIV testing and prevention counselors conducted the VCTR with detainees who were aged 13–19 years and reported being sexually active. Study recruitment, assent, data collection, and testing protocols and procedures were approved by the Institutional Review Boards of Henry Ford Health System, Wayne State University, and the Wayne County JDF. The sample for this analysis includes JDF residents that were tested by HFHS staff during the study period.

#### Measures

##### Demographics

Information on detainee date of birth, ethnicity, race, and sex at birth were collected via self-report from assenting detainees and recorded on the VCTR form.

##### Domestic violence and forced sex

Domestic violence and forced sex appear as check boxes on the VCTR form under the section entitled “situational co-factors”, and for this analysis were defined as an affirmative answer to these items during the VCTR interview.

##### Sexual risk behaviors

Sexual risk behaviors appear as check boxes on the VCTR form under the sections entitled “situational co-factors”, and “sex partner risk”. For this analysis, sexual risk behaviors were defined as an affirmative response to the following questions: sex with an anonymous partner, sex without a condom, sex with a person of unknown HIV status, sex under the influence of alcohol, and sex under the influence of marijuana. We created the variable “transactional sex” which comprised an affirmative answer to paying for sex, providing sex for drugs or money, or commercial sex work, listed in the situational co-factors section.

##### History of chlamydia

A history of chlamydia infection was defined as an affirmative response to questions about previous diagnosis of chlamydia. Chlamydia was selected because of its high prevalence among juvenile detainees (Spaulding et al., 2013)

#### Analysis

All analyses were conducted with Statistical Analysis System (SAS) version 9.1. The frequency and proportion of adolescents reporting factors associated with sexual risk behaviors and domestic violence or forced sex are presented. Bivariate analysis included chi-square tests for statistical significance, along with odds ratios and corresponding 95% confidence intervals. Fisher’s exact test was used when at least 25% of expected cell counts in the table were < 5. Logistic regression analysis was used to describe the association of domestic violence and forced sex (independent variables) and sexual risk factors (dependent variables) using adjusted odds ratios (95% confidence intervals). Each model controlled for age, race, ethnicity, and sex. Given the number of statistical tests, the risk of type I error was reduced by setting our significance level to *p* < 0.01, while *p* values > 0.01 < 0.05 are interpreted as suggestive of an association.

## Results

### Characteristics of the study cohort

**Fig. 1:**
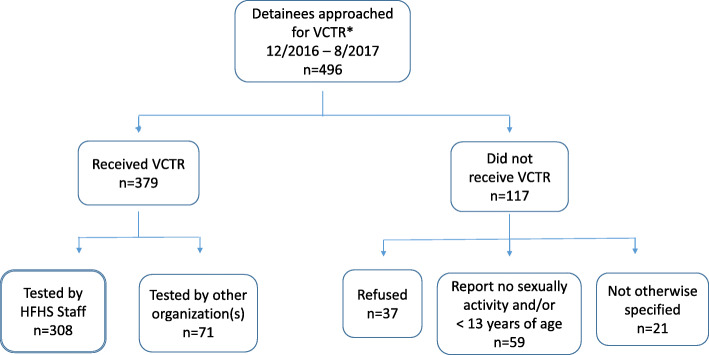
Flow diagram showing breakdown of study population. * Voluntary Counseling, Testing and Referral for sexually transmitted diseases

In total, 496 JDF residents were approached for VCTR (Fig. 1). Of those, 379 (76.4%) assented and received testing. HFHS tested a total of 308/379 (81.3%) youth (our analytic sample) while the remaining 71 youth were tested by another organization whose data was not available for this analysis. Of the 117 that did not receive VCTR, 37/117 (31.6%) refused, and 59/117 (50.4%) reported no sexual activity or were not yet 13 years of age (our IRB was only approved for youth 13 and older). Information was lacking on the remaining 21/117 (17.9%). The present analysis is based on the 308 individuals tested by HFHS. The mean age of the sample was 16.1 years (SD ± 1.2) and ranged from 13 to 19 years (Table 1). The sample was 77.3% male. The ethnic distribution of the group was 75.3% African American, 12.3% White, 8.8% Hispanic, and 3.6% other. Nearly two-thirds of the detainees (64.5%) had residential zip codes within the city of Detroit.

**Table 1:** Characteristics of a sample of juvenile detainees participating in Voluntary Counseling Testing and Referral (*n* = 308)

Age mean (±SD), range	16.1 (±1.22), 13–19 years	
	n	%
**Sex**		
Male	238	77.3
Female	70	22.7
**Race**		
African-American	232	75.3
White	38	12.3
Hispanic	27	8.8
Other	11	3.6
History of chlamydia	30	9.7
**Sexual risk behaviors**		
Sex with an anonymous partner	70	22.7
Sex without a condom	194	63.0
Sex with a person of unknown HIV status	126	40.9
Sex under the influence of alcohol	69	22.4
Sex under the influence of marijuana	153	49.7
**Self-report of trauma**		
Domestic violence only	13	4.2
Forced sex only	12	3.9
Both	6	1.9

### Prevalence of sexual risk behavior and a history of chlamydia

A history of chlamydia infection was reported by 30 (9.7%) of youths in the sample (24.3% in females and 5.5% in males). The proportion of youth self-reporting sexual risk behaviors was as follows: 194 (63%) reported having sex without a condom, 126 (40.9%) reported having sex with a person of unknown HIV status, 70 (22.7%) reported having sex with an anonymous partner, 69 (22.4%) reported having sex under the influence of alcohol and 153 (49.7%) reported having sex under the influence of marijuana (Table 1). Overall, 31 respondents (10%) reported exposure to domestic violence or forced sex (Table 1). Of these, 12 (3.9%) reported a history of forced sex only, 13 (4.2%) reported an exposure to domestic violence only, and 6 (1.9%) reported experiencing both.

### Association of age, race/ethnicity, and sex to sexual risk behavior, domestic violence and forced sex

We examined age, race/ethnicity, and sex in relation to sexual risk behavior, a history of chlamydia, and domestic violence and forced sex (self-report of trauma). Sexual risk behaviors by age are shown in Table 2. Youth reporting sex without a condom (*p* <  0001), sex with a partner of unknown HIV status (*p* <  0.001) and sex under the influence of alcohol (*p* <  0.0001) were significantly older than youth who did not report these sexual risk behaviors. There were no significant age differences by report of domestic violence or forced sex (Table 2). African Americans were significantly less likely than Whites to report sex under the influence of alcohol, Odds Ratio (95% confidence interval) = 0.27 (0.13, 0.55), *p* <  0.001 (see Table E1 in Supplement), but no other associations with race/ethnicity (including self-report of domestic violence and forced sex) met criteria for statistical significance (Table E1 in Supplement). When examining risk behaviors by sex (Table 3), females were more likely to report a history of chlamydia infection than males, OR = 5.55, (2.54, 12.13); *p* < 0.0001, and were also more likely to report forced sex than males, OR = 14.62 (4.63, 46.13); Fisher’s *p* < 0.0001.

**Table 2:** Report of history of chlamydia, sexual risk behaviors and self-report of domestic violence or forced sex (trauma) by mean age among juvenile detainees participating in Voluntary Counseling, Testing, and Referral (*n* = 308)

	Yes			No			***p*** value
	mean	(sd)	Range	mean	(sd)	Range	
History of chlamydia	16.58	(1.00)	14.47–18.64	16.04	(1.23)	13.03–19.58	0.020
**Sexual risk behavior**							
Sex with an anonymous partner	16.39	(1.20)	13.28–18.64	16.00	(1.22)	13.03–19.58	0.020
Sex without a condom	16.36	(1.16)	13.18–19.58	15.64	(1.20)	13.03–18.38	< 0.0001
Sex with a person of unknown HIV status	16.37	(1.09)	13.08–18.64	15.90	(1.27)	13.03–19.58	< 0.001
Sex under the influence of alcohol	16.67	(1.12)	13.18–18.90	15.92	(1.20)	13.03–19.58	< 0.0001
Sex under the influence of marijuana	16.26	(1.17)	13.08–18.90	15.92	(1.25)	13.03–19.58	0.014
**Self-report of trauma**							
Domestic violence	16.58	(1.13)	14.47–18.26	16.06	(1.22)	13.03–19.58	0.074
Forced sex	16.27	(0.98)	13.98–17.67	16.08	(1.24)	13.03–19.58	0.53

**Table 3:** Report of history of chlamydia, sexual risk behaviors and self-report of domestic violence and forced sex (trauma) by sex among a sample of juvenile detainees participating in Voluntary Counseling, Testing, and Referral (*n* = 308)

	Female (***n*** = 70)		Male (***n*** = 238)		OR^**a**^	(95% CI)	***p*** value
	n	(%)	n	(%)			
History of chlamydia	17	(24.3)	13	5.5	5.55	(2.54, 12.13)	< 0.0001
**Sexual risk behavior**							
Sex with an anonymous partner	14	(20.0)	56	(23.5)	0.81	(0.42, 1.57)	0.536
Sex without a condom	50	(71.4)	144	(60.5)	1.63	(0.91, 2.91)	0.092
Sex with person of unknown HIV status	28	(40.0)	98	(41.2)	0.95	(0.55, 1.64)	0.860
Sex under the influence of alcohol	23	(32.9)	46	(19.3)	2.04	(1.13, 3.70)	0.017
Sex under the influence of marijuana	38	(54.3)	115	(48.3)	1.27	(0.74, 2.17)	0.380
**Self-report of trauma**							
Domestic violence	9	(12.9)	10	(4.2)	3.36	(1.31, 8.64)	0.019 ^b^
Forced sex	14	(20.0)	4	(1.7)	14.62	(4.63, 46.13)	< 0.0001^b^

^a^ Odds ratio (95% Confidence Interval); ^b^Fisher’s Exact Test

### Association of domestic violence and forced sex to sexual risk behavior

Results of logistic regression are shown in Table 4. Using *p* < 0.01 as criteria for statistical significance, and after controlling for age, race/ethnicity, and sex, report of domestic violence was significantly associated with sex under the influence of alcohol, OR = 6.57 (2.25, 19.17), *p* < 0.001; and sex under the influence of marijuana, OR = 9.18 (2.03, 41.51); *p* = 0.004. Forced sex was significantly associated with having sex with a person of unknown HIV status, OR = 6.92 (2.05, 23.41), *p* < 0.001.

**Table 4:** Logistic regression results for the association of domestic violence and forced sex (trauma) to history of chlamydia and sexual risk behaviors among a sample of juvenile detainees participating in Voluntary Counseling, Testing and Referral

	Independent variables			
	Domestic violence		Forced Sex	
Dependent variables	aOR^**a**^ (95% CI)	***p***-value	aOR (95% CI)	***p***-value
History of chlamydia	3.73 (1.17, 11.99)	0.027	2.69 (0.81, 8.94)	0.107
Sex with an anonymous partner	2.65 (0.98, 7.19)	0.055	2.91 (0.97, 8.73)	0.057
Sex without a condom	1.85 (0.55, 6.26)	0.324	2.41 (0.62, 9.38)	0.203
Sex with a person of unknown HIV status	3.05 (1.09, 8.55)	0.034	6.92 (2.05, 23.41)	< 0.001
Sex under the influence of alcohol	6.57 (2.25, 19.17)	< 0.001	2.73 (0.91, 8.17)	0.073
Sex under the influence of marijuana	9.18 (2.03, 41.51)	0.004	1.43 (0.50, 4.04)	0.504

^a^Odds ratio adjusted for age, race, and sex

## Discussion

This study explored the independent relationship of forced sex and domestic violence to sexual risk behaviors and previous chlamydia infection among a group of detained youth in an urban detention facility. We found significant associations between domestic violence and sex under the influence of alcohol or marijuana, while forced sex was associated with having sex with a person of unknown HIV status. While our study design does not permit definitive statements about causality, our results and those of others suggest that domestic violence and forced sex represent traumatic experiences that can lead to sexual risk behaviors. Both domestic violence and forced sex are among the 10 experiences included among ACES or adverse childhood experiences (Wolff & Baglivio, 2017).

Comparing our results with the literature on the association of domestic violence to sex under the influence of alcohol or marijuana was not straightforward. The term domestic violence has several synonyms in the literature including family violence (Fix, Alexander, & Burkhart, 2018) family dysfunction (Dube et al., 2003), household dysfunction (Campbell, Walker, & Egede, 2016) and household violence (Naramore et al., 2017). The definition varies considerably in the literature and across states in the US (Child Welfare Information Gateway, 2018). Related to this point, is that some definitions of domestic violence include intimate partner violence, which may represent a variation in the context of the violence experienced or witnessed by the youth (Child Welfare Information Gateway, 2018). Moreover, “experiencing domestic violence” could involve being the victim or the victimizer, although both fall within the purview of the definition used by the National Council of Juvenile and Family Court Judges (Child Welfare Information Gateway, 2018).

There is a robust literature on the relationship of ACES (which include family violence) to substance use among juvenile detainees. Charak, Ford, Modrowski, and Kerig (2019) latent class analysis showed that youth in the two highest risk groups, violent environment and polyvictimization, had a higher likelihood of alcohol and drug problems (Charak et al., 2019). It is unlikely that these relationships are linear. According to the literature, the association between family violence and sex under the influence of alcohol or marijuana is impacted by race (Fix et al., 2018) depression (Gillman et al., 2018; Schumacher et al., 2018) and sex (Dembo et al., 2018; Dembo, Krupa, Wareham, Schmeidler, & DiClemente, 2017; Morais, Alexander, Fix, & Burkhart, 2018). Of note is that Craig, Intravia, Wolff, and Baglivio (2019), found that with low substance use, the relationship between family violence and recidivism was no longer significant (Craig et al., 2019). Finally, our assessment of the literature focused on domestic violence/family violence and did not venture into intimate partner violence. A relationship of intimate partner violence and sexual risk behavior may represent another, and different, pathway in the association of traumatic experiences to sexual risk behavior among juvenile detainees.

Our finding that forced sex was associated with sex with a partner of unknown HIV status seems aligned with recent literature showing specific trauma (e.g., maltreatment) or ACES (which includes sexual abuse) associated with sexual risk behavior (e.g., unprotected sex) (K. Clements-Nolle et al., 2017; Fasula et al., 2018; Leve et al., 2015). In a sample of justice-involved females, Leve et al. (2015) found a significant association between child maltreatment (sexual abuse or physical abuse as determined by caseworker report of documentation) and unsafe sexual practices, and the subsequent likelihood of developing an STI (Leve et al., 2015). Moreover, the association was moderated by the female participant’s comfort at communicating with her partner, such that the association of maltreatment was stronger at lower levels of comfort. Similar results were reported by Clements-Nolle et al. (2017) with a sample of female juvenile offenders in which report of child maltreatment was associated with unprotected sex (K. Clements-Nolle et al., 2017). This relationship was mediated by substance use (K. Clements-Nolle et al., 2017). Our results are likely driven by females in the dataset who are more likely than males to report both a history of domestic violence and forced sex.

A sexual partner with unknown HIV status does include persons engaged in transactional sex. In a study of over 64,000 justice-involved youth, those arrested for trading sex were significantly more likely to have a higher ACE score, and to have a higher prevalence of one or more of the 10 ACES than youth arrested for other reasons (Naramore et al., 2017). These youth are particularly vulnerable to re-victimization, implying that interventions designed to break the cycle of delinquency and victimization could have a substantial impact on the life trajectory of these youth (Naramore et al., 2017; Negriff, 2018; Thompson et al., 2017).

There are several conceptual models that provide likely explanations for the association of trauma and sexual risk behavior among detainees. In a discussion of trauma and high-risk behavior, Kianpoor and Bakhshani (2012) proposes the concept of avoidance through dissociation: a separation of the mental processes that are usually integrated into a person’s conscious awareness (Kianpoor & Bakhshani, 2012). Substance use becomes an adaptive response in an attempt to avoid or block awareness of painful experiences. Likewise, the concept of “chemical dissociation” describes the use of alcohol or drugs to escape the emotional distress as a result of trauma (Kianpoor & Bakhshani, 2012).

A model developed by Ford, Chapman, Mack, and Pearson (2006), posits that traumatic experiences overwhelm the executive function of the brain (Ford et al., 2006). Over time, dysregulation of thoughts, emotions, and behaviors results in formation of “rigid cognitive schemas” or inflexible patterns of thinking and processing information (Ford et al., 2006). This process could cause affected youth to learn fewer coping strategies, exhibit lack of impulse control, and lose the ability to self-regulate, demonstrated perhaps through sexual risk behaviors. Youth experiencing “dysregulated emotions and survival or victim-based information processing” may appear defiant and angry as a means of self-protection from further victimization (Ford et al., 2006).

Negriff (2018) provided evidence for a developmental cascade (i.e., chain reaction, snowball effect (Masten & Cicchetti, 2010) from maltreatment to risk behavior (peer delinquency, substance use, delinquency, sexual behavior). Maltreatment had significant direct effects on all risk behaviors, and although the authors found several developmental paths leading from maltreatment to risk behaviors, the results supported a developmental sequence from maltreatment to sexual behavior (especially early initiation of sex), to peer delinquency, and culminating in substance use (Negriff, 2018).

The Negriff (2018) study was conducted among youth from Child Protective Services. Evidence of this sequence can aid in identifying youth at risk and subsequently delivering a more focused intervention (Negriff, 2018). More studies of this nature are needed to further examine causality, temporal linkages, and potential pathways from trauma to sexual risk behavior among juvenile detainees, as well as the factors mediating or moderating those pathways (Scott-Sheldon et al., 2016). Maltreatment can have long-term effects, according to the literature, and has been shown to predict outcomes in adulthood including employment status, substance use, and criminal arrest (Barnert et al., 2016; Negriff, 2018). Detention may represent an opportunity to intervene at a critical juncture in the lives of the youth presenting to the facility (Barnert et al., 2016).

### Limitations

There are several important limitations. The prevalence of trauma is low in our population compared to other reports. Baglivio (2016) reported a prevalence of “family violence” of 84% and 81% respectively for female and male juvenile offenders (Baglivio, 2016; Dembo et al., 2019). This is much higher than our prevalence of 6.1%. Moreover, we only asked about two traumatic events, and others have shown that justice-involved youth are typically exposed to more than one type of trauma (Charak et al., 2019; Kerig, 2018). If we missed (misclassified) trauma exposure among youth reporting sexual risk behaviors, the potential misclassification bias would be differential and likely biased toward the null (Rothman & Greenland, 1998). The low prevalence of trauma, the potential for misclassification, and the fact that our analysis is based on a single detention facility, suggests that caution is needed when comparing our results to that of other studies.

Other limitations include potential selection and measurement bias. Only data from those detainees tested by HFHS were available for analysis. This limited dataset, coupled with the low prevalence of trauma mentioned above, indicates we may have a population that is less representative of all detainees. The data in this analysis are based on self-report obtained through interviews of the juvenile detainees during VCTR and as a result it is possible that participants may have been reluctant to disclose past exposure or current behaviors. The VCTR form used did not have survey items with psychometric properties but uses check boxes to indicate respondent answers. This form may have left room for subjective interpretation by the respondent and the person performing the HIV testing and counseling. Questions about sex with an “anonymous partner” on the VCTR form were worded differently than in other studies e.g., Rosengard et al. (2006), used the phrasing “sex with someone you did not know well”, while others have used “casual sex” or “non-romantic partners” (Grello, Welsh, Harper, & Dickson, 2003). The Youth Risk Behavior Surveillance System (YRBSS) asks if respondents used a condom during last intercourse (43.1%) (Kann et al., 2016), while a study of juvenile detainees in Cook County asks how many juvenile detainees had unprotected vaginal sex in the past month (35%) (Teplin, Mericle, McClelland, & Abram, 2003). In a study among juvenile detainees in Hillsborough County, Florida, youth were categorized as “seldom or never use condoms” (15.3%) (Belenko et al., 2008). These differences in questionnaire wording challenged our ability to make direct comparison to previous literature. Widespread adoption of national and standardized questionnaires in studies of adolescent sexual behavior would facilitate comparisons of adolescent populations, and specifically, justice-involved youth, across studies.

Other limitations include the lack of information on socioeconomic status which is an important determinant of involvement in the juvenile justice system, and the cross-sectional design of this study. This design does not allow for determination of temporality, i.e., identifying domestic violence or forced sex as predictors of sexual risk behavior. Despite these limitations, we found significant associations that are aligned with findings of previous researchers (Dembo et al., 2018; Dembo, Krupa, et al., 2019; Moreland et al., 2018).

In summary, the literature is clear in the need for trauma-informed interventions targeting sexual risk behaviors among detainees (Senn, Carey, & Vanable, 2008). According to a review of the 2020 CDC Compendium of Evidence Based Interventions designed to reduce the risk of HIV, only 2 of the 16 interventions listed were conducted with detained youth (Centers for Disease Control and Prevention, 2020). Advocating for better screening and healthcare in detention facilities, experts suggest that failure to address the needs of these youth impacts the communities and families to which they return (Barnert et al., 2016; Belenko, Dembo, Rollie, Childs, & Salvatore, 2009; Dembo, Faber, et al., 2019). Effective interventions designed to improve sexual health of justice-involved youth should target the interrelated domains of trauma, substance use, and sexual risk behavior (Skinner-Osei et al., 2019).

## Conclusions

In our analysis, exposure to domestic violence was related to sex under the influence of marijuana and sex under the influence of alcohol. Forced sex was related to sex with a partner of unknown HIV status. These behaviors increase the risk of STI in an already vulnerable population for whom health care is often intermittent at best. Justice-involved youth experience more trauma than youth in the general population. Conceptual models suggest that trauma is the cornerstone of sexual risk behavior and substance use, threatening problems that extend throughout adulthood for justice-involved youth (Barnert et al., 2016; Tull, Weiss, & McDermott, 2016). While substantial challenges to addressing the needs of juvenile detainees exist, juvenile justice reform must incorporate trauma-informed interventions and policies to improve the life.

trajectories of justice-involved youth (Scott et al., 2018; Skinner-Osei et al., 2019).

## Supplementary information

**Additional file 1: Table E1.** Report of history of chlamydia, risky sexual behaviors, and traumatic experiences by race/ethnicity (n = 308).

## Data Availability

The datasets used and/or analyzed during the current study are available from the corresponding author upon reasonable request.

## Abbreviations

ACEs: Adverse Childhood Experiences
HFHS: Henry Ford Health System
HIV: Human Immunodeficiency Virus
JDF: Juvenile Detention Facility
SAS: Statistical Analysis System
STI: Sexually Transmitted Infection
VCTR: Voluntary Counseling Testing and Referral
YRBSS: Youth Risk Behavior Surveillance System
