# Supplementary material for: Self-report of domestic violence and forced sex are related to sexual risk behaviors in a sample of juvenile detainees
Source: Health Justice. 2020 Jun 23;8:15. doi: 10.1186/s40352-020-00116-4 (PMC7313184; doi:10.1186/s40352-020-00116-4)
Supplement: Supplementary file 1 — Additional file 1: Table E1. Report of history of chlamydia, risky sexual behaviors, and traumatic experiences by race/ethnicity (n = 308). [file 40352_2020_116_MOESM1_ESM.docx]

| **E1. Report of history of chlamydia, risky sexual behaviors, and traumatic experiences by race/ethnicity** (n =308) | | | | |
| --- | --- | --- | --- | --- |
| **Risk group** | **n positive** | **%** | **OR (95% CI)** | **p-value** |
|  |  |  |  | |
| White | 38 | (12.3) | *Reference* | |
| History of chlamydia | 2 | 5.3 |  |  |
| Sex with an anonymous partner | 8 | 21.1 |  |  |
| Sex without a condom | 19 | 50.0 |  |  |
| Sex with a person of unknown HIV status | 17 | 44.7 |  |  |
| Sex under the influence of alcohol | 17 | 44.7 |  |  |
| Sex under the influence of marijuana | 17 | 44.7 |  |  |
| Domestic violence | 5 | 13.2 |  |  |
| Forced sex | 4 | 10.5 |  |  |
| African-American | 232 | (75.3) |  | |
| History of chlamydia | 26 | 11.2 | 2.27 (0.52, 9.99) | 0.392 |
| Sex with an anonymous partner | 55 | 23.7 | 1.17 (0.50, 2.69) | 0.720 |
| Sex without a condom | 150 | 64.7 | 1.83 (0.92, 3.65) | 0.084 |
| Sex with a person of unknown HIV status | 97 | 41.8 | 0.89 (0.44, 1.77) | 0.735 |
| Sex under the influence of alcohol | 41 | 17.7 | 0.27 (0.13, 0.55) | <0.001 |
| Sex under the influence of marijuana | 111 | 47.8 | 1.13 (0.57, 2.26) | 0.722 |
| Domestic violence | 13 | 5.6 | 0.39 (0.13, 1.17) | 0.150 |
| Forced sex | 13 | 5.6 | 0.50 (0.16, 1.64) | 0.274 |
| Hispanic | 27 | (8.8) |  | |
| History of chlamydia | 2 | 7.4 | 1.44 (0.19, 10.91) | 1.000 |
| Sex with an anonymous partner | 5 | 18.5 | 0.85 (0.25, 2.96) | 0.801 |
| Sex without a condom | 18 | 66.7 | 2.00 (0.72, 5.56) | 0.181 |
| Sex with a person of unknown HIV status | 7 | 25.9 | 0.43 (0.15, 1.26) | 0.121 |
| Sex under the influence of alcohol | 9 | 33.3 | 0.62 (0.22, 1.72) | 0.355 |
| Sex under the influence of marijuana | 18 | 66.7 | 2.47 (0.89, 6.88) | 0.081 |
| Domestic violence | 1 | 3.7 | 0.25 (0.03, 2.31) | 0.388 |
| Forced sex | 1 | 3.7 | 0.33 (0.03, 3.10) | 0.393 |
| Other* | 11 | (3.6) |  | |
| History of chlamydia | 0 | 0.0 | 0.63 (0.03, 14.2) | 1.000 |
| Sex with an anonymous partner | 2 | 18.2 | 0.83 (0.15, 4.65) | 1.000 |
| Sex without a condom | 7 | 63.6 | 1.75 (0.44, 6.98) | 0.425 |
| Sex with a person of unknown HIV status | 5 | 45.5 | 1.03 (0.27, 3.96) | 1.000 |
| Sex under the influence of alcohol | 2 | 18.2 | 0.27 (0.05, 1.44) | 0.165 |
| Sex under the influence of marijuana | 7 | 63.6 | 2.16 (0.54, 8.64) | 0.270 |
| Domestic violence | 0 | 0.0 | 0.26 (0.01, 5.17) | 0.574 |
| Forced sex | 0 | 0.0 | 0.33 (0.02, 6.68) | 0.562 |
|  | | | | |
| *includes: Arab/Chaldean, Asian, Hawaiian/Pacific Islander, Native American | | | | |
